# Literature Review and Clinical Presentation of Bilateral Acetabular Fractures Secondary to Seizure Attacks

**DOI:** 10.1155/2012/240838

**Published:** 2012-09-02

**Authors:** Alexandre H. Nehme, Jihad F. Matta, Alaa G. Boughannam, Fouad C. Jabbour, Joseph Imad, Ramzi Moucharafieh

**Affiliations:** Department of Orthopedic Surgery and Traumatology, Saint Georges University Medical Center, University of Balamand, P.O. Box 166378, Achrafieh, Beirut 1100 2807, Lebanon

## Abstract

Central acetabular fracture dislocation is usually caused by high-energy external trauma. However, 26 cases that occurred as a result of a seizure attack appeared in the literature from 1970 to 2007, with the seizure attacks themselves caused by many different factors. In this setting, the central acetabular fracture not caused by direct trauma might initially remain unnoticed leading to a delayed diagnosis. In some cases, this may lead to death as a result of massive blood loss. We here present a case of bilateral central acetabular fracture dislocation as a result of a seizure attack.

## 1. Introduction

Acetabular fracture dislocations are common and typically result from high-energy external trauma, as observed in patients after high-speed motor vehicle accidents or after a fall with direct impact [1]. However, 26 cases of acetabular fractures occurring as a result of seizure attacks appeared in the literature since 1970 [2–26]. Therefore, it is quite remarkable that the muscle contractions during a seizure are sometimes of sufficient force to induce such a fracture.

In this setting, the central acetabular fracture not caused by direct trauma might initially remain unnoticed leading to a delayed diagnosis [26]. In some cases, death might occur as a result of massive blood loss [11]. We here present a case of bilateral central acetabular fracture dislocation as a result of status epilepticus in a patient with a history of Von Hippel-Lindau disease.

## 2. Case Report

A 68-year-old Caucasian male was admitted to the internal medicine ward of our hospital for status epilepticus. The patient's medical history included Von Hippel-Lindau disease with multiple cysts in the liver, pancreas, both kidneys and a surgically excised cerebellar hemangioblastoma treated 25 years ago, with the insertion of a Ventriculoperitoneal shunt. He also suffered from epilepsy, recurrent urinary tract infections, chronic renal failure, recurrent atrial flutter, high blood pressure, and Parkinson disease with delirium. His current medication included valproic acid for epilepsy and a combination of trandolapril and verapamil for high blood pressure. 

**Figure 1:**
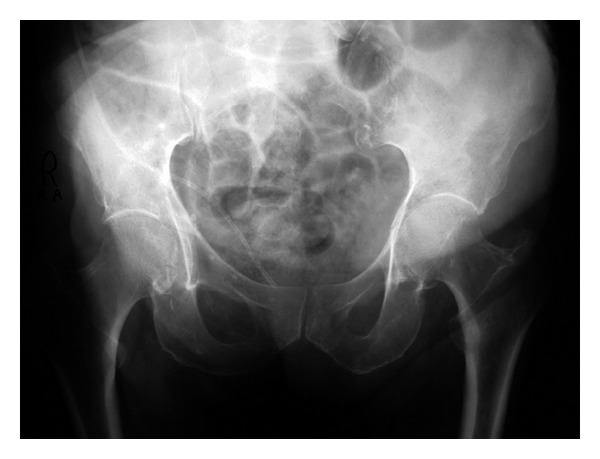
Linear non displaced fracture of the left acetabulum.

In the emergency department (ED) a computed tomography (CT) scan of the head was done and revealed no new abnormalities, and an anteroposterior pelvic X-ray showed a linear nondisplaced fracture of the left acetabulum which remained unnoticed initially (Figure 1). After performing the pelvic X-ray and during his stay in the ED, the patient experienced several similar episodes of epilepsy but of shorter duration so the patient was started on diazepam, midazolam, and valproic acid. For airway protection, the patient was intubated and transferred to the intensive care unit where he was stabilized.

**Figure 2:**
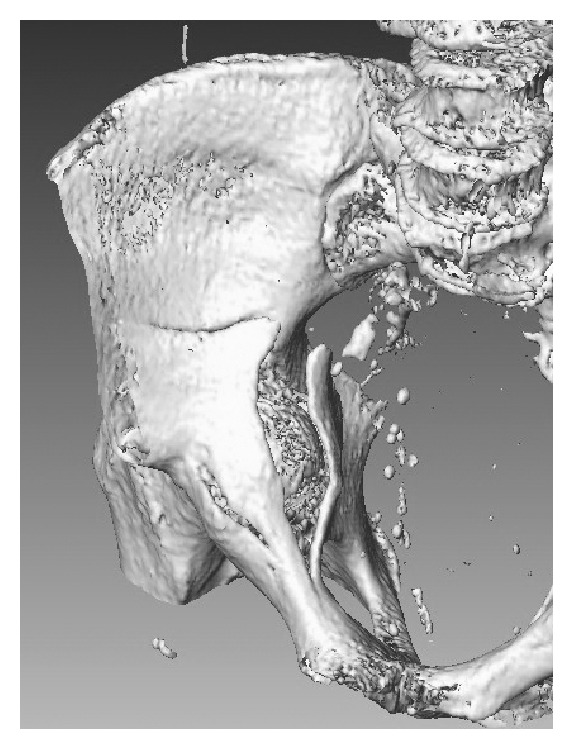
Intrapelvic 3D CT reconstructed view of the right Hip with protrusion of the right femoral head.

**Figure 3:**
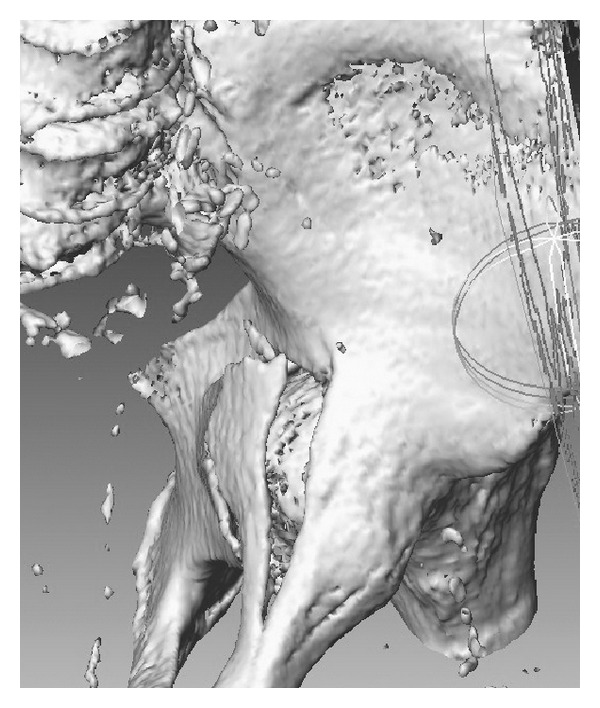
Intrapelvic 3D CT reconstructed view of the right Hip with protrusion of the left femoral head.

**Figure 4:**
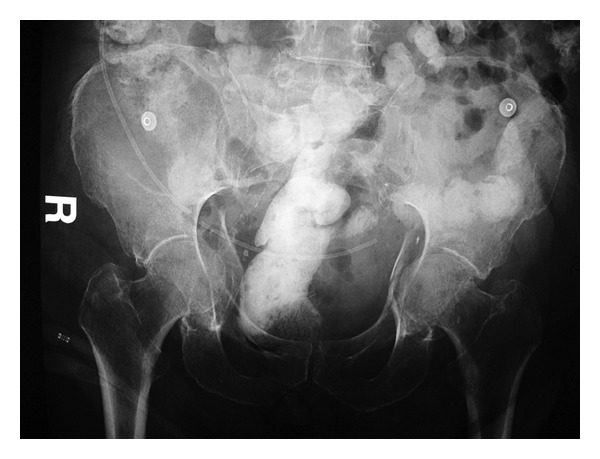
AP Pelvis X-ray showing displaced fractures of both acetabuli with intrapelvic protrusion of both femoral heads.

After two days, the patient was transferred to the internal medicine ward. During this time, the patient was unresponsive, somnolent, or sedated. It was noticed that the patient's blood hemoglobin level was decreasing gradually from 9.8 to 8.1 g/dL over a period of 48 hours. Transfusion of 3 units of concentrated human red blood corpuscles was performed, and his treating internist ordered a CT scan of the abdomen to search for a cause that was thought to be an intracystic hemorrhage. By chance, displaced fractures of both acetabuli with intrapelvic protrusion of both femoral heads were discovered (Figures 2 and 3). Another anteroposterior pelvis X-ray was subsequently done and confirmed the intrapelvic displacement and protrusion (Figure 4).

**Figure 5:**
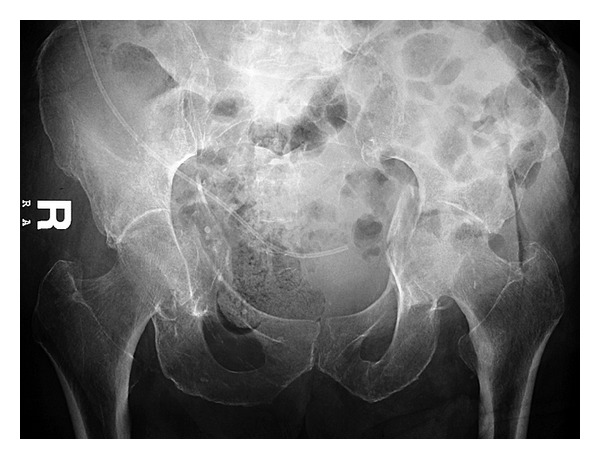
Acceptable hip congruity achieved following traction and formation of a callus with adequate healing of both acetabuli.

Nonoperative treatment was selected because the patient continued to experience mild-to-severe seizures in spite of the antiepileptic drugs. Therefore, bilateral transcondylar traction pins were applied to allow femoral skeletal traction of 8 kg on each lower extremity. The patient remained non-weight-bearing for 3 months. Traction was interrupted at 6 weeks after injury and range of motion exercises of both hips was started and increased gradually. Pain in both hips gradually diminished, and acceptable congruity was achieved based on X-ray findings. Later followup radiography showed the formation of a callus with adequate healing of both acetabuli (Figure 5).

Several attempts of full weight bearing with 3 persons support and a walker were unsuccessful at three months, following the injury because the patient developed severe postimmobilization amyotrophy in spite of daily physiotherapy and muscle strengthening exercises. Moreover, in spite of adequate nursing care, the patient developed deep sacral ulcers and died 4 months after his injuries from septic shock. 

## 3. Discussion

Orthopedic injuries associated with or resulting from convulsions are not uncommon. On rare occasion, tonic-clonic seizure activity has been reported to cause acetabular fracture dislocations [2–26]. Usually Acetabular fracture dislocations result from external trauma such as motor vehicle collisions or fall from a height with direct impact [1], but given the tremendous mass of pelvitrochanteric muscle acting in a craniomedial direction, it is understandable that forceful contractions during generalized tonic-clonic seizure activity can also result in a fracture dislocation. The mechanism of injury could be explained by massive uncontrolled muscle contractions which can force the head of the femur in the craniomedial direction against the acetabulum [26]. Moreover, patients with repetitive seizures can be considered with a higher risk for central acetabular fracture dislocations, as seen in our case where the first X-ray done in the emergency department showed only a linear nondisplaced fracture. The severely displaced bilateral central acetabular fracture dislocations occurred only after repetitive uncontrolled seizures in spite of medications.

It is also imperative to mention that long-term seizure patients who are under antiepileptic medications affecting intestinal calcium absorption can suffer from anticonvulsant osteopathy [27], which might increase their susceptibility to fracture.

Operative treatment for central acetabular fractures dislocation was reported in some articles [23, 24]. Total hip arthroplasty was performed for the nonunion cases or the comminuted fractures. Nonoperative treatment was selected for our case because of the patient's medical history, and current medical status with continuous seizures.

## 4. Conclusion

The current case provides an example of a rare and relatively unknown but life-threatening fracture pattern caused by a seizure attack. Late diagnosis of central acetabular fractures may lead to sudden death due to massive blood loss. The mortality rate of such fractures in all reported cases (including our case) is 18.5% (5/27). Hence, the possibility of acetabular fracture dislocation should be kept in mind when examining a postictal patient.
